# Mitigating the Impact of Cellulose Particles on the Performance of Biopolyester-Based Composites by Gas-Phase Esterification

**DOI:** 10.3390/polym11020200

**Published:** 2019-01-24

**Authors:** Grégoire David, Nathalie Gontard, Hélène Angellier-Coussy

**Affiliations:** JRU IATE 1208—CIRAD/INRA/Montpellier Supagro/University of Montpellier, 2 Place Pierre Viala, Bat 31, CEDEX 01, F-34060 Montpellier, France; gregoire.david@supagro.fr (G.D.); nathalie.gontard@inra.fr (N.G.)

**Keywords:** poly(hydroxybutyrate-*co*-valerate) (PHBV), biocomposite, gas-phase esterification, cellulose, water transfer

## Abstract

Materials that are both biodegradable and bio-sourced are becoming serious candidates for substituting traditional petro-sourced plastics that accumulate in natural systems. New biocomposites have been produced by melt extrusion, using bacterial polyester (poly(3-hydroxybutyrate*-co-*3-hydroxyvalerate)) as a matrix and cellulose particles as fillers. In this study, gas-phase esterified cellulose particles, with palmitoyl chloride, were used to improve filler-matrix compatibility and reduce moisture sensitivity. Structural analysis demonstrated that intrinsic properties of the polymer matrix (crystallinity, and molecular weight) were not more significantly affected by the incorporation of cellulose, either virgin or grafted. Only a little decrease in matrix thermal stability was noticed, this being limited by cellulose grafting. Gas-phase esterification of cellulose improved the filler’s dispersion state and filler/matrix interfacial adhesion, as shown by SEM cross-section observations, and limiting the degradation of tensile properties (stress and strain at break). Water vapor permeability, moisture, and liquid water uptake of biocomposites were increased compared to the neat matrix. The increase in thermodynamic parameters was limited in the case of grafted cellulose, principally ascribed to their increased hydrophobicity. However, no significant effect of grafting was noticed regarding diffusion parameters.

## 1. Introduction

Cellulose is the most abundantly available renewable polymer on earth [1]. Its use as a filler in polymer matrices for the production of biocomposites has interested scientists and industries for decades, and even more so now with the will for developing new sustainable, biodegradable, light, and functional materials [2,3,4,5,6,7,8,9,10]. Compared to common industrial fillers, such as glass or carbon fibers, cellulose presents huge advantages, including its renewable character, low density, low cost, large availability throughout the world, non-abrasive behavior towards process equipment, high stiffness and tensile strength, and full biodegradability under natural conditions [10,11]. Unfortunately, cellulosic fillers also display some drawbacks due to their strong polar character, giving rise to three major limitations when used in composite materials. The first one is their poor compatibility with hydrophobic polymer matrices generally used, resulting in weak interfacial adhesion. The final properties of composite materials strongly depend on the intrinsic properties of the filler and matrix, and on the interface area, i.e., the compatibility between the two constituents. The quality of the filler–matrix interface is crucial since the load transfer from the matrix to the filler should be efficient enough to allow the material to stand up to the external mechanical solicitation [12]. The second one is their poor dispersion state within aforesaid matrices, due to the formation of cellulose aggregates through hydrogen bondings. The third limitation is associated with their strong sensitivity to water and even moisture, which induce swelling and loss of mechanical properties under ageing conditions [12,13]. To overcome these limitations, cellulosic fillers are generally submitted to various surface modifications to minimize the interfacial energy between the fillers and non-polar polymer matrix. These modifications present an opportunity for developing biocomposites with new functional properties.

The literature describes several strategies for developing bicomposites, including physical and chemical treatments [13,14,15,16,17,18,19]. Among them, esterification with fatty acids, from hexanoic (C6) to dodecanoic acids (C22), is a common treatment applied to decrease the surface hydrophilicity of cellulose fibers used in composite materials [20,21,22,23,24,25]. Le Moigne et al. [12] reported that in most studies, chemical surface functionalization of natural fibers enhanced the mechanical properties of biocomposites. Concerning pure cellulosic reinforcements, Pasquini et al. showed that their surface modification with octadecanoyl and dodecanoyl chloride in heterogeneous conditions led to improved interfacial adhesion with a polyethylene-based matrix, a higher filler dispersion level, and better water resistance [21]. However, mechanical performances of biocomposites were not improved due to the degradation of cellulose with the treatments. In a study by Freire et al. [22], where they focused on the impact of the degree of substitution (DS) and fatty chain length on the properties of acylated cellulose low-density polyethylene-based composites. The study showed that water resistance, interfacial adhesion, and mechanical properties were enhanced, especially for composites displaying low DS-modified cellulose. De Menezes et al. [23] detected an increased dispersion state of esterified cellulose whiskers, and improved elongation at break with increasing lengths of the grafted chains.

With the objective of developing greener processes of cellulose surface modification, a gas-phase esterification with palmitoyl chloride was previously studied [26]. This process was adapted from work by Berlioz et al. and Fumagalli et al. [27,28], who in both cases used this reagent derived from one of the most common fatty acids of vegetable origin. The occurrence of the chemical modification of micrometric cellulose particles was evidenced by solid-phase ^13^C NMR, with DS values ranging between 0.01 and 0.14. It was shown, by contact angle measurements, that the treatment made cellulose particles drastically more hydrophobic, without altering their bulk properties, i.e., morphology, crystallinity, and thermal stability.

Mechanical and thermal properties of esterified cellulose-based biocomposites have been already investigated and presented in some literature reviews [12,16,29], however, very few studies are available on mass transfer properties in such biocomposites [30,31,32,33,34]. When mass transfers are evaluated, it is essentially at the macroscopic scale via permeability measurements. Little interested is focused on the evaluation of diffusion and sorption phenomena, which are absolutely necessary to correctly formalize the structure/mass properties relationships. Furthermore, to the best of our knowledge, transfer properties of both moisture and liquid water in esterified cellulose composites have not been yet investigated. Besides, research still needs to ascertain these materials by selecting appropriate polymer matrices. In order to benefit from the fully biosourced and biodegradable character of cellulose, increasing interest is being given to biopolymers that are biosourced and fully biodegradable in natural conditions, i.e., polyhydroyalakanoates (PHAs). Among PHAs, poly(hydroxybutyrate-*co*-valerate) (PHBV) is a very promising polymer, because its easily processable and displays similar properties to common polyolefins [32,35,36,37,38,39,40,41,42].

In this context, the objective of the present study was to investigate the effects of gas-phase esterification of cellulose on the functional properties of PHBV/cellulose biocomposites. For that purpose, PHBV/cellulose biocomposites with different filler contents were prepared by melt extrusion using either virgin or gas-phase esterified cellulose. Changes in thermal stability, tensile properties, water vapor, and liquid water transfer properties of biocomposites were discussed in relation to some molecular structural parameters, including PHBV molecular weight and crystallinity, and to the microstructure of materials qualitatively assessed by SEM observations.

## 2. Materials and Methods

### 2.1. Materials

Cellulose, in powder form, was supplied by Arbocel J. Rettenmaier & Söhne (Rosenberg, Germany) under the reference Arbocel^®^ (grade BE 600-10 TG). Cellulose particles were characterized by a true density of 1.59 g·cm^−3^, a cellulose content of 99.5%, and a median apparent diameter (d_50_) of 18 µm. In a previous study dealing with exactly the same grade of cellulose [43], particles were assimilated to cylinders with the length and diameter corresponding to the major and the minor axis, respectively. These two shape descriptors were determined in volume, and the median values were respectively 32 ± 2 µm and 19 ± 1 µm.

PHBV was purchased from NaturePlast (Ifs, France) under the reference PHI 002. As reported by the manufacturer, PHBV contained 1–3 mol % of valerate and had a true density of 1.24 g·cm^−3^.

### 2.2. Methods

#### 2.2.1. Grafting of Cellulose

**Figure 1:**
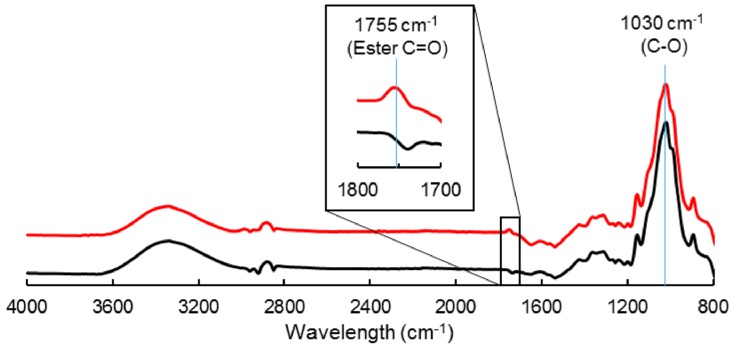
FT-IR spectra of C-virgin (**‒**) and C-grafted (**‒**).

Cellulose particles were subjected to a gas-phase esterification using palmitoyl chloride, as described by David et al. [26]. After a drying step at 60 °C overnight, the reaction was conducted in a 2 L reactor, on a 100 g batch at 100 °C, and 2 mbar during 15 h. The cellulose particles were inserted in nylon mesh bags, which were positioned on a Teflon grid above the liquid reagent. The reagent was used in excess compared to surface hydroxyl groups (0.2 eq compared to total anhydroglucose units). A nitrogen flow was used to evacuate the reaction by-products that were vacuum-pumped. The resulting grafted cellulose sample was purified using acetone and finally dried at 60 °C. The grafted cellulose (noted C-grafted in the following article) was characterized by a degree of substitution (DS) of 0.02, as measured by ^13^C solid-state NMR spectroscopy [26]. The virgin cellulose, without treatment, was noted C-virgin. The covalent grafting of cellulose by esterification was evidenced by FT-IR analysis (ATR-FTIR, VERTEX 70v Bruker, Ettlingen, Germany), with the appearance of the ester carboxyl signal at 1745 cm^−1^ together with the intensity decrease of the hydroxyl parts (3000–3600 cm^−1^) (Figure 1). FT-IR spectra were normalized with respect to the peak height at 1030 cm^−1^, considered as an invariant for the cellulose backbone.

#### 2.2.2. Preparation of Composite Materials

Composite films displaying an average thickness of 300 µm were prepared using a lab-scale twin screw extruder, with a L/D ratio of 40 and a screw diameter of 16 mm (Eurolab, Thermo Scientific, Karlsruhe, Germany), equipped with a flat die of 300 µm of thickness and a calendering unit. Raw PHBV pellets and cellulose particles (either virgin or esterified) were previously dried at 60 °C overnight before extrusion. Cellulose and PHBV pellets were introduced with a loss-in-weight twin screw feeder (Brabender, Duisburg, Germany) and a volumetric single screw feeder (Brabender), respectively. The temperature profile from the feeding to the die varied from 80 °C to 180 °C (80–160–160–160–160–170–170–180–180–160 °C). The screw speed was set to 300 rpm and the total flow rate was 1.0 kg·h^−1^. The average residence time was 4.0 min. Three filler contents were produced, i.e., 10, 20, and 33 wt %, respectively named PHBV-10VC, PHBV-20VC and PHBV-33VC, for composite with virgin cellulose as filler or PHBV-10GC, PHBV-20GC and PHBV-33GC for composite containing grafted cellulose.

#### 2.2.3. Characterization of Films

For each sample, film thickness was systematically measured using a precision gauge (Hanatek-model FT3, East Sussex, UK) on at least five different positions. Thickness mean values were considered for all following calculations (mechanical properties, water vapor sorption kinetics, water vapor permeability, and liquid water uptake). Samples were stored in a hermetic drum at 23 °C in presence of silica gel (around 0% RH) before further analysis.

A gel permeation chromatograph GPC PL-50 Plus system equipped with two columns of 300 mm PL-gel 5 µm mixed-C (200–2,000,000 g·mol^−1^) (Polymer Laboratories, Church Stretton, UK), and a refractive index detector was used to measure the molecular weight of PHBV. The eluent was chloroform, the flow was set at 1.0 mL·min^−1^ and the volume of injection was 20 µL. PHBV samples (10 mg) were previously dissolved in 2 mL of chloroform in a closed tube under stirring at 50 °C. Samples were filtered on Macherey–Nagel Chromafil Xtra syringe filter (PTFE-45/25, Düren, Germany) with 0.45 µm pore size. The GPC equipment was calibrated with polystyrene standards. To measure the broadness of the molecular weight distribution of the matrix, the polydispersity index (Ip) was calculated as follows:

(1) $$\mathrm{Ip}=\frac{Mw}{Mn}$$

Differential scanning calorimetry (DSC) analysis was carried out using a thermo-modulated calorimeter (Q200 modulated DSC, TA Instruments, New Castle, DE, USA). Aluminium pans (Tzero Aluminium Hermetic pan, TA Instruments New Castle, DE, USA) were filled with approximately 10 mg of sample and hermetically sealed. Analyses were performed in triplicate. The purge gas was nitrogen, with a flow rate of 50 mL·min^−1^. Each sample was first heated up to 200 °C at 10 °C·min^−1^, then cooled at 10 °C·min^−1^ until temperatures reached −30 °C, and finally heated again from −30 °C to 200 °C at a heating rate of 10 °C·min^−1^. The resultant thermogram displayed the variation of heat flow per gram of sample (W·g^−1^) towards temperature (°C). From this thermogram, crystallization temperature (T_c_) and melting temperature (T_m_) were measured respectively from peaks of the cooling ramp and second heating ramp, respectively. Melting enthalpy (ΔHm) was calculated from the area under the peak observed on the 1st and 2nd heating ramps. ∆H^0^_m_ = 146 J·g^−1^ was taken for 100% crystalline PHBV matrix from [44], and *w* is the weight fraction of the matrix in the composite calculated from TGA analysis. Crystallinity of the materials was calculated as follow (Equation (2)):

(2) $$\mathrm{Xc}=\left(\frac{\Delta H_{m}}{\Delta H^{0}{}_{m}}\right)\times \left(\frac{100}{w}\right)$$

Thermogravimetric analysis (TGA) under nitrogen flow (50 mL·min^−1^) was carried out using a Mettler TGA2 apparatus (Schwerzebbach, Switzerland) equipped with a XP5U balance (precision of 0.0001 mg). For each measurement, about 40 mg of materials were used, and the heating rate was 10 °C·min^−1^ from 25 °C to 800 °C. The maximum degradation temperature (T_deg_) corresponded to the temperature at which the degradation rate was maximum. The onset and offset degradation temperatures (T_onset_ and T_offset_, respectively) were measured respectively when the first derivative of the weight loss became higher than 0.1 %·°C^−1^ and lower than 0.1 %·°C^−1^. Analyses were done in triplicate. The weight filler content of the composite was determined from inflection point between 280 °C and 300 °C corresponding to the end of PHBV degradation and beginning of cellulose degradation. To correct the overlapping thermal degradation, standard curves for virgin and grafted cellulose in PHBV were used (R^2^ = 0.999). The volume filler content was deduced from the weight filler content based on knowledge of the true density of both biocomposite constituents.

Scanning electron microscopy (SEM) observations were performed with a S-4800 microscope (Hitachi, Japan) after coating the sample with Pt by cathode pulverization. In case of cryo-fractured section observations, the specimens were frozen in liquid nitrogen then fractured before coating.

Tensile tests. Mechanical properties were evaluated through tensile tests conducted at room temperature by a texture analyzer (Zwick BZ5/TN1S, Metz, France) on dog-bone shaped film specimens (width of 4 mm and gauge length of 45 mm). The specimens were previously stored in a closed chamber at 23 °C and 50% RH. Stress–strain curves obtained with a cross-head speed of 1 mm·min^−1^ helped determine Young’s modulus (E), nominal stress at break (σ), and nominal strain at break (ε). The energy at break was calculated from the total area under the stress–strain curve. Ten replicates were realized for each formulation (10 wt %, 20 wt %, and 33 wt %).

Water vapor sorption kinetics were measured at 20 °C using a controlled atmosphere micro-balance (DVS, Surface Measurement System Ltd., London, UK). The mass evolution of the material was recorded using a Cahn D-200 microbalance with a precision of 0.1 µg. The relative humidity was also followed over time. A pre-drying step was first run at 60 °C in an oven, then the sample was dried over P_2_O_5_ in a desiccator, and finally placed in the DVS equipment at 0% RH for 5 h at 20 °C. In the case of cellulose, 1 mg of cellulose was deposited in an aluminum pan (DSC Tzero^®^ pans provided by TA Instruments), which was placed in the DVS nacelle, as previously described by Thoury et al. [45]. In the case of biocomposite films, circular pieces of a 7.5 mm diameter were cut and deposited directly in the DVS nacelle. Increasing relative humidity steps (0, 20, 40, 60, 80, and 95%) were performed for the same sample, and each step time was adjusted to insure equilibrium. Water vapor sorption isotherms were determined from the equilibrium moisture contents at each RH step. Tests were performed at least in duplicate.

Water vapor permeability (WVP) were gravimetrically determined at 23 °C using an adapted ASTM E96/E96M procedure. Discs of films (five repetitions) were sealed in permeation cells filled with distilled water that were put into a desiccator containing silica gel. A relative humidity (RH) gradient equal to 0–100% was obtained (i.e., ΔP = 2809 Pa at 23 °C, assuming that RH on the silica gel is negligible). The permeation area was 9.08 cm^2^. Periodic weightings determined the rate of water vapor movement through the films. WVP (mol·s^−1^·Pa^−1^·m^−1^) values were calculated from Equation (3), where S is the slope of the weight change from the straight line (g·h^−1^), A is the permeation area (m^2^), t is the average specimen thickness (m), Psat is the saturation vapor pressure at 23 °C (Pa), and M_H2O_ is the molar mass of water (g·mol^−1^).

(3) $$\mathrm{WVP}=\frac{S\times t}{3600\times A\times \mathrm{Psat}\times M_{H_{2}O}}$$

Liquid water uptake kinetics were measured on discs of 25 mm diameter cut from composite films. After drying overnight at 60 °C, the specimens were weighted using a balance with ±0.001 mg precision and then immersed into distilled water at 20 °C. At various time intervals, the samples were removed, blotted to remove free water on their surface, and immediately weighed using an analytical balance. Experiments were performed in triplicate until reaching the water uptake equilibrium for each sample.

#### 2.2.4. Modeling

Diffusion in an infinite plane sheet was used to describe water vapor diffusion of fillers, matrix, and composite. It was assumed that the diffusion in the material was isotropic and independent of time and space. In addition, the possible swelling of the sample with increasing relative humidity was considered negligible. The apparent diffusion coefficient was estimated using the analytical solutions provided by Crank [46]. Considering an isotropic diffusion, the diffusion can be reduced to a pure axial diffusion that occurs in an infinite plane sheet of thickness *L* (m). The diffusion equation at time *t*, at position *z*, and for film of thickness *L* is given in Equation (4), where *D* (m^2^·s^−1^) corresponds to the water’s apparent diffusivity in the material.

(4) $$\frac{\partial C}{\partial t}\left(t,z\right)=D\left(\frac{\partial^{2}C\left(t,z\right)}{\partial z^{2}}\right)$$

In case of water vapor sorption (deposition of the sample in a DVS pan), the plane sheet was insulated at its bottom, and defined by z∈[0, L], with initial and boundary conditions given in Equation (5).

(5) $$C\left(t=0,z\right)=C_{0}\;\forall \;z\in \;\left[0,L\right]\;\frac{\partial C}{\partial z}\left(t,z=0\right)=0\;\forall \;t\geq 0\;C\left(t,z=L\right)=C_{\infty }\;\forall \;t\geq 0$$

In case of liquid water sorption (immersion of the samples in water), it was considered that the plane sheet was not insulated and that it was defined by $z\in \left[\frac{-L}{2},\frac{L}{2}\right]$. Equation (4) was kept by considering boundary conditions given in Equation (6).

(6) $$C\left(t=0,z\right)=C_{0}\;\forall \;z\in \;\left[\frac{-L}{2},\frac{L}{2}\right]\;\frac{\partial C}{\partial z}\left(t,z=0\right)=0;\;\forall \;t\geq 0\;C\left(t,z=\pm \frac{L}{2}\right)=C_{\infty };\;\forall \;t\geq 0$$

The analytical solution for an infinite plane sheet of thickness *L* (m) in both cases was described by Equation (7) [46]:

(7) $$\frac{M_{t}}{M_{\infty }}=1-\overset{\infty }{\underset{n=0}{\sum }}\frac{8}{{\left(2n+1\right)}^{2}\pi^{2}}exp\left(\frac{-D{\left(2n+1\right)}^{2}\pi^{2}t}{4L^{2}}\right)$$

where $M_{t}$ and $M_{\infty }$ denote, respectively, the water mass uptake at time *t* and the corresponding value for infinite time.

Water’s apparent diffusivity D was determined using the lsqnonlin function that solved the corresponding nonlinear least squared problem in MATLAB^®^ R2015b software. The idea was to minimize the root mean square error (RMSE) between experimental sorption kinetics and simulated ones, as shown in Equation (7). N is the number of experimental data points from DVS, m_sim_(t) and m_exp_(t) are, respectively, the estimate and the experimental mass uptake at time t. Simulations were performed by using Equation (8).

(8) $$\mathrm{RMSE}=\sqrt{\frac{\sum_{i=1}^{N}{\left(m_{\mathrm{sim}}\left(t_{i}\right)-m_{\exp}\left(t_{i}\right)\right)}^{2}}{N}}$$

## 3. Results and Discussion

### 3.1. Impact of Gas-Phase Esterification on Some Macromolecular Parameters of PHBV

Functional properties of biocomposites, e.g., mechanical and mass transfer properties, are known to be strongly dependent on the polymer’s macromolecular parameters, including mainly molecular weight and crystallinity, that influence properties of cellulose grafting on PHBV.

#### 3.1.1. Molecular Weight

Table 1 shows PHBV’s molecular weight after processing by melt extrusion. The introduction of cellulose fillers resulted in a very slight decrease in the polymer’s molecular weight, without a significant effect of grafting or filler content. It could be concluded that the thermal degradation of polymer chains was not promoted by the presence of cellulosic particles, either virgin or grafted, as already described for plasticized PHBV [47] or lignocellulosic-based biocomposites [48]. In any case, the molecular chains of PHBV are still considered as long chains because they are larger than 150 kDa [49].

#### 3.1.2. Differential Scanning Calorimetry

A heat-cool-heat cycle was performed using differential scanning calorimetry (DSC) in order to investigate the crystallization behavior of “as produced” composites, as well as the intrinsic crystallization behavior after having erased the thermal history of materials. Virgin PHBV (extruded under the same conditions as biocomposites) presented a crystallinity degree (Xc) of 73 ± 1% (measured during the second heating ramp). As previously reported [48], this high crystallinity was ascribed to the addition of boron nitride as a nucleating agent (0.5 wt %) in the commercial formulation (Table 1). This value was in agreement with results reported in previous studies using the same grade of PHBV [50]. It is worth noting that lower crystallinity values were found in the work of Berthet et al. [48,49], probably due to the differences in processing conditions and lower molecular weight. The addition of cellulosic fillers, either virgin or grafted, decreased the crystallinity degree to 66 ± 1% for all samples, except for PHBV-20VC for which the crystallinity remained constant. This decrease could be attributable to a hindered motion of the polymer segments due to the presence of fillers. Fillers could interact with the matrix or act as local defects, inhibiting the growth of PHBV crystals. The fact that the cristallinity of PHBV-20VC remained constant would indicate that the mobility of polymer chains was not affected. In the present study, the little effect of filler on crystallinity could be explained by the presence of boron nitride that masked the potential nucleating effects of cellulose [41].

Regarding the melting temperature, it was not significantly impacted by the addition of either virgin or esterified cellulose [21,22], even for high filler contents, which could be due to the unchanged polymer molecular weight. However, the crystallization temperature significantly decreased with the addition of esterified cellulose, while it remained unchanged in the case of virgin cellulose. This highlighted that the presence of fatty acids on the surface of cellulose, which inhibited the initiation of the crystallization growth of the surrounding matrix.

#### 3.1.3. Thermal Stability

**Figure 2:**
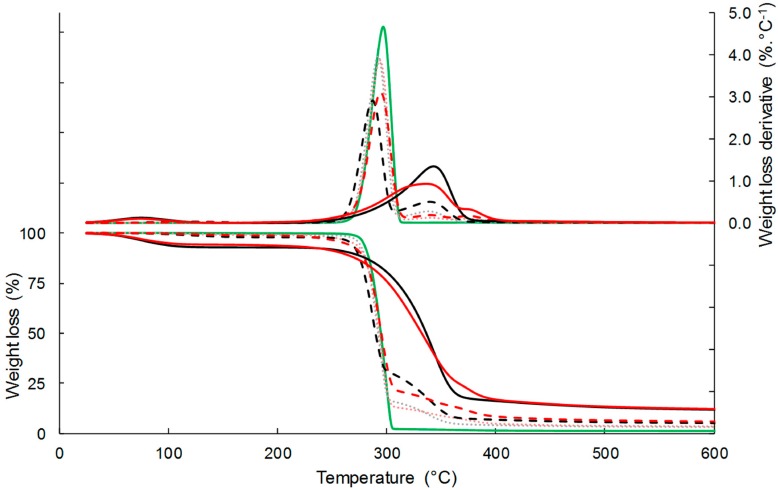
TG and DTG curves of C-virgin (**‒**), C-grafted (**‒**), PHBV (**‒**), PHBV-20CV (**···**), PHBV-20CG (**···**), PHBV-33CV (**-** **-**), and PHBV-33CG (**--**) under N_2_.

The thermal stability of the composites and their separated constituents was examined by thermogravimetric analysis (TGA) under inert atmosphere (Figure 2). Neat PHBV was characterized by one main sharp thermal degradation occurring between 260 °C and 310 °C, due to the chain scission reaction mechanism. The temperature at the maximal rate of degradation (T_deg_) was 297 °C (Table 2). The degradation of virgin cellulose and esterified cellulose occurred on a larger temperature range than that of PHBV, with T_deg_ of respectively 343 °C and 336 °C. The temperature range was even more important for esterified cellulose, with T_onset_ and T_offset_ values of 247 °C and 398 °C, respectively, against 259 °C and 375 °C for virgin cellulose. As already showed by David et al. [26], the earlier thermal degradation of grafted cellulose could be ascribed to the high lability of ester bonds. It is worth noting that a second degradation peak of low intensity was observed on the first derivative curve (DTG) for grafted cellulose (around 375 °C), which was related to the palmitoyl moiety David et al. [26]. The weight loss around 100 °C, which was observed for both C-virgin and C-grafted, was due to the water loss contained in the samples.

TGA curves of composite materials showed two degradation steps, the first one corresponding to the degradation of the PHBV matrix, and the second one to the filler degradation, with a small overlap. The introduction of cellulose fillers resulted in a slight decrease in the thermal stability of PHBV, which was attributed to the interactions between cellulose and PHBV, as already reported in Reference [41]. The thermal degradation of cellulose produced small polar molecules that likely facilitated breaking of PHBV chains. This negative effect was even more important by increasing the content of virgin cellulose, while the filler content had no impact in the case of esterified cellulose. Indeed, T_deg_ decreased from 296.8 °C for the neat PHBV down to 294.4 °C for PHBV-33GC, and 287.1 °C for PHBV-33CV. The second degradation corresponding to cellulose decomposition was much less steep for composites filled with esterified cellulose than with virgin cellulose. It was shown that this effect could not be explained only by a simple rule of mixing the respective effects of each constituent, meaning that the degradation of grafted cellulose was slowed while embedded in the PHBV matrix. It was thus assumed that the interactions at the filler/matrix interface could also play a role [22]. Regarding temperatures at the maximal rate of degradation, differences between materials filled with virgin cellulose or grafted cellulose were only significant for a filler content of 10 wt %. Nevertheless, it is worth noting that T_deg(2)_ of composites filled with grafted cellulose were not any lower than T_deg(2)_ of composites filled with virgin cellulose. This phenomenon could be explained by the fact that stronger filler/matrix interactions occurred in the case of composites filled with grafted cellulose, thereby hindering the thermal degradation of the filler. This has been previously observed by Freire et al. [22].

### 3.2. Impact of Gas-Phase Esterification on Interfacial Adhesion: Qualitative Evaluation

**Figure 3:**
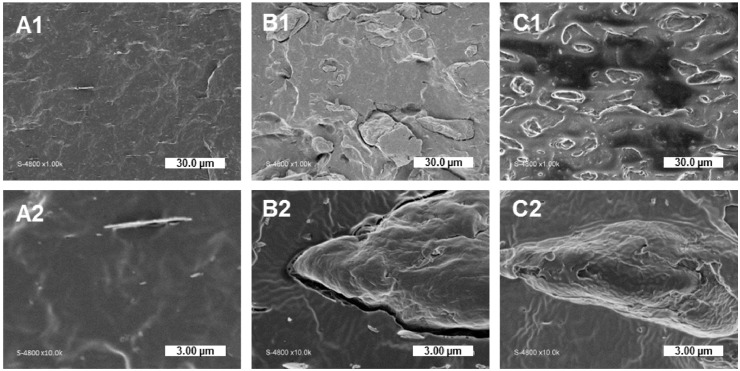
SEM pictures of cryo-fractured sections: (**A1**,**A2**) neat PHBV, (**B1**,**B2**) PHBV-20VC, and (**C1**,**C2**) PHBV-20CG.

The impact of gas-phase esterification on the filler/matrix interfacial adhesion was qualitatively assessed by SEM observations of cryo-fractured cross-sections (Figure 3). Neat PHBV displayed a smooth surface with the inclusion of boron nitride used as a nucleating agent (Figure 3A). In the case of virgin cellulose, the distinction between cellulose particles and the matrix was obvious, with clear gaps at the filler/matrix interface (Figure 3B). In the case of composites filled with grafted cellulose, fillers were intimately embedded in the matrix, with particles perfectly coated by the polymer (Figure 3C). Such an improved wetting of fillers by the surrounding PHBV matrix would be ascribed to the increased hydrophobicity of grafted cellulose, as previously demonstrated by contact angle measurements in previous work by David et al. [26]. Contrary to Pasquini et al. [21] who obtained similar results for LDPE filled with esterified cellulose, an improvement of the filler’s dispersion state was not significantly observed in the present study.

### 3.3. Impact on Water Transfer Properties in Resulting Composites

Moisture and liquid water transfer properties of biocomposites are important aspects that must be assessed, because they govern many other functional properties of usage conditions, including material stability. They are particularly important when materials are used for packaging applications since they ensure the preservation of packed goods that are sensitive to hydration or dehydration.

#### 3.3.1. Water Vapor Sorption Kinetics

**Figure 4:**
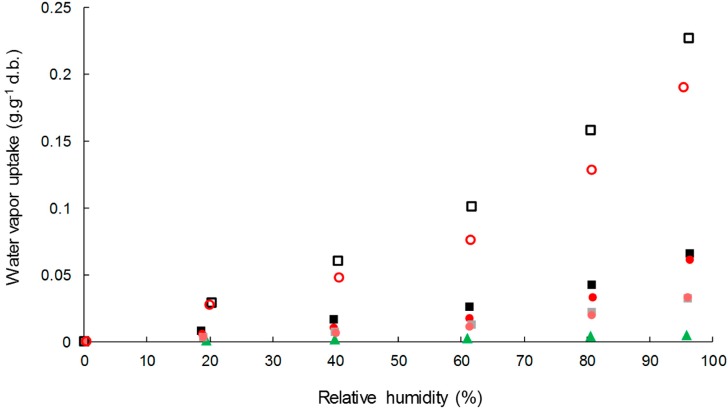
Water vapor sorption isotherm of C-virgin (**□**), C-grafted (o), PHBV (▲), PHBV-20VC (■), PHBV-20GC (●), PHBV-33VC (■) and PHBV-33GC (●).

Water vapor sorption kinetics at successive relative humidity (RH) steps allowed the assessment of moisture sorption isotherms and estimating moisture’s apparent diffusivity. Water vapor sorption isotherms of cellulose samples, neat PHBV, and biocomposite films are shown in Figure 4. Cellulose absorbed much more water vapor than PHBV, with moisture uptake at the equilibrium at 95% of RH of 0.5 ± 00 g·g^−1^ d.b., 22.7 ± 0.0 g·g^−1^ d.b., and 19.0 ± 0.2 g·g^−1^ d.b. for PHBV, virgin cellulose, and esterified cellulose, respectively. This corroborated the high hydrophobic character of PHBV compared to cellulose samples, even esterified. In the case of cellulose samples, a classical sigmoidal shape was observed for water vapor isotherms. The region until 20% of RH was ascribed to the absorption of a monolayer of water onto specific sites of the material’s surface, with no difference in sorption behavior between the two samples of cellulose. Then, a linear portion between 20% and 60% RH corresponded to the dissolution of water vapor in the materials due to the porous structure of cellulose and to the stacking of water layers. Finally, for high relative humidity the water vapor uptake increased abruptly due to the formation of water cluster and capillarity [51]. As previously reported, esterification hindered water sorption behavior on the two last zones, due to the modification of pore volume of cellulose particles [26].

The addition of cellulose particles in PHBV led to a significant increase in water vapor sorption, with water vapor uptake at 95% of RH of 6.6 ± 0.0 g·g^−1^ d.b. and 6.1 ± 0.0 g·g^−1^ d.b. for PHBV-33VC and PHBV-33GC, respectively.

In order to understand the contribution of each constituent in the composites, experimental data were compared to values predicted using a simple rule of mixture by considering weight fractions (w) and the water vapor content (M) of each constituent at each RH (Equation (9)).

(9) $$M_{\mathrm{composite}}=M_{\mathrm{filler}}\times w_{\mathrm{filler}}+M_{\mathrm{matrix}}\times w_{\mathrm{matrix}}$$

**Figure 5:**
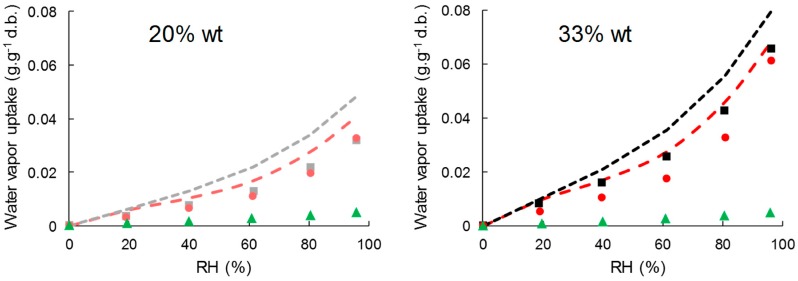
Simulated sorption isotherms from Equation (9) for PHBV-based composites: PHBV (▲), PHBV-20VC (**- -**), PHBV-20GC (**- -**), PHBV-33VC (**- -**), PHBV-33GC (**- -**).

It was shown that the rule of mixture over-estimated the water sorption in the biocomposites for the two types of fillers, especially for high RH (Figure 5). It can be concluded that water vapor sorption of composites was not a simple addition of the contribution of cellulose and PHBV, as already shown by Wolf et al. [34] in PHBV/wheat straw fiber composites. It is worth noting that the slight decrease of crystallinity would have been in favor of a higher water vapor uptake. Furthermore, since no significant changes in PHBV’s molecular weight was evidenced by GPC, such results could be explained by structural changes of the polymer matrix not evidenced in the present paper, e.g., the formation of rigid amorphous regions within the PHBV matrix [52], or by hindered water vapor sorption of cellulose due to the surrounding matrix.

**Figure 6:**
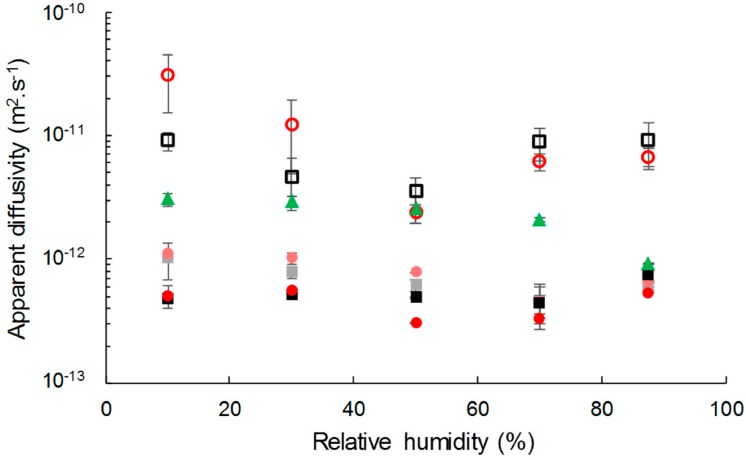
Apparent diffusivity of C-virgin (**□**), C-grafted (o), PHBV (▲), PHBV-20VC (■), PHBV-20GC (●), PHBV-33VC (■) and PHBV-33GC (●).

Apparent diffusivity values were identified at each RH step by fitting a mathematical model to the experimental data from water vapor sorption kinetics (Figure 6). Apparent diffusivity of water vapor was lower in composites than in the neat matrix, even though the two types of cellulose displayed higher diffusivity values. The diffusivity slightly decreased with the filler content. This unexpected behavior was also reported by Wolf et al. [34] for PHBV/wheat straw biocomposites. This could be ascribed to a tortuosity effect that would be emphasized by the formation of a barrier interphase.

Grafting did not affect the water vapor diffusivity in the composite, but had an impact on the diffusivity of the insulated cellulose. For relative humidity lower than 50% of RH, the diffusion coefficient of water in grafted cellulose was higher than in virgin cellulose. It could not be explained by a difference in crystallinity since it has been shown in a previous paper that it was not affected [26]. For RH higher than 50%, the diffusivity of water vapor in grafted cellulose became lower than in virgin cellulose, probably due to a water clustering effect emphasized by the hydrophobic character of esterified cellulose, as already observed in hydrophobic polymers [53]. A recrystallization phenomenon of the cellulose hindered by the grafting could also be possible.

It is worth noting that for a given filler content, moisture’s apparent diffusivity values were the same for composites filled with grafted or virgin cellulose. The difference in behavior between grafted and virgin cellulose was annihilated when they were incorporated in PHBV.

#### 3.3.2. Water Vapor Permeability (WVP)

Water vapor permeability (WVP) of the PHBV matrix was found to be 3.7 ± 1.0 × 10^−13^ mol·m^−1^·s^−1^·Pa^−1^, which was slightly lower than other values mentioned in the literature [48,54]. This difference could be explained by a small change in valerate content. The incorporation of cellulose, either virgin or grafted, increased WVP up to 36 ± 3.2 × 10^−13^ mol·m^−1^·s^−1^·Pa^−1^ for PHBV-33VC (Table 3). Knowing that the permeability coefficient combines the effects of diffusion and solubility according to the relation P = D × S, the increase in WVP could be ascribed to an increase in diffusion (kinetic parameter) and/or solubility (thermodynamic parameter), with the possibility of competitive effects. In the present study, we can conclude that WVP of cellulose-based biocomposites was governed by a solubility phenomenon since the effect of the previously demonstrated decreased diffusion was drastically compensated by the increased solubility.

The increase in WVP was limited by esterification for high filler contents. For a filler content of 33 wt %, esterification reduced WVP by more than a factor of two compared to virgin cellulose. This could be ascribed to the slight decrease in the diffusion parameter, induced by a possible better dispersion state of fillers and filler/matrix adhesion, but more likely to the decreased solubility.

Water vapor permeability (WVP) is a key feature for food packaging. Obtaining a range of WVP values is very interesting since it could fulfill the requirements of different kinds of food products. As an example, composites with high filler contents could be used for packaging respiring products.

#### 3.3.3. Liquid Water Absorption

**Figure 7:**
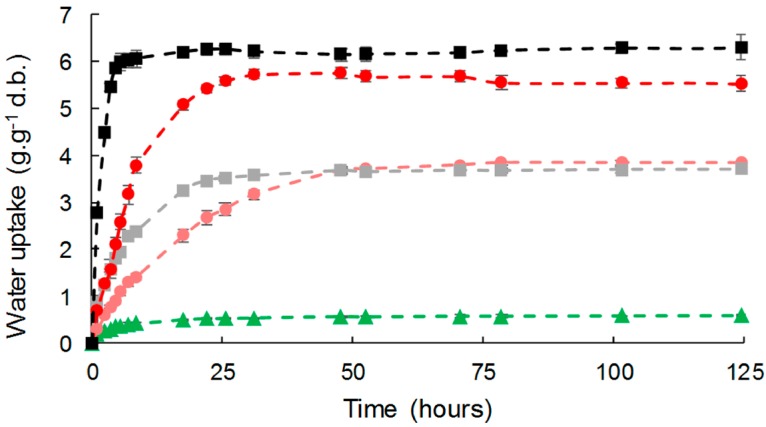
Water liquid sorption kinetics for PHBV-based composites: PHBV (▲), PHBV-20VC (■), PHBV-20GC (●), PHBV-33VC (■), PHBV-33GC (●). Points were linked for more visibility.

Liquid water absorption was assessed as a function of time for the different composites (Figure 7). Water was absorbed by all the composites during the experiments following a two-step pattern, with significant differences in both the initial diffusion rate and water uptake at equilibrium. A fast water uptake step preceded an equilibrium plateau suggesting a Fickian behavior.

PHBV, due to its hydrophobic nature, showed low liquid water uptake with a value at equilibrium of 0.58 g·g^−1^ d.b. The introduction of cellulose fillers led to an increase in water uptake, which was proportional to the filler content, as already described for cellulose in LDPE or PLA [22,55]. As observed for WVP, the effect of gas-phase esterification was only significant for high filler content (33 wt %). It is worth noting that liquid water uptakes at equilibrium were similar to those of moisture at 95% of RH (Table 3).

The time to reach the equilibrium was around 50 h for all materials, this duration being related to the thickness of the films (around 300 µm in the present study). However, a slight but significant decrease in apparent diffusivity was noticed in the case of biocomposites, which was in agreement with the results obtained for moisture (Table 3). This could be ascribed to an increased diffusion pathway induced by the introduction of filler. For a virgin cellulose content of 33 wt %, this tortuosity phenomenon competed with the formation of cellulose aggregates and poor interfacial adhesion that would be in favor or water diffusion. Logically, esterification induced a reduction of apparent diffusivity.

### 3.4. Impact on Gas-Phase Esterification on Mechanical Properties of the Resulting Composites

**Figure 8:**
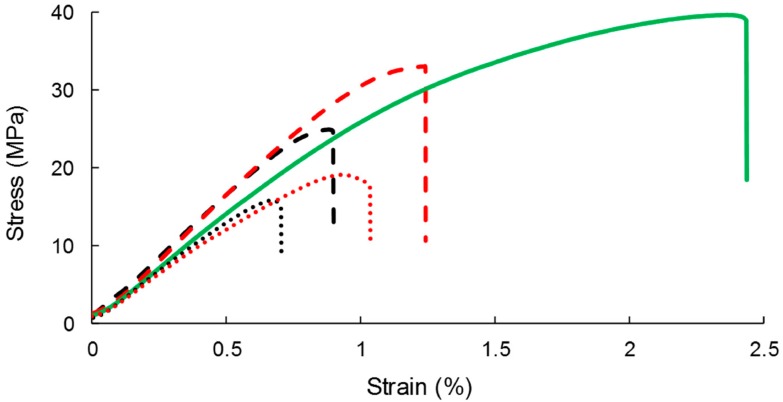
Representative stress–strain curves of the composites materials: PHBV (**‒**), PHBV-10VC (**- -**), PHBV-10GC (**- -**), PHBV-33VC (···), PHBV-33GC (···).

PHBV-based composites displayed the common behavior of a rigid and fragile thermoplastic, as previously observed in similar studies [48,50] (Figure 8). The neat PHBV matrix was characterized by a Young’s modulus of 2.9 ± 0.1 GPa, a stress at break of 39.7 ± 0.1 MPa, and strain at break of 2.4 ± 0.2% (Table 4). The Young’s modulus was not deeply impacted by the incorporation of cellulose. An increase in Young’s modulus with the filler content could be expected when the rigidity of fillers is higher than the matrix’s [56,57,58,59], which is not the case in the present study [48]. The low aspect ratio of the studied cellulose particles could explain this phenomenon.

The incorporation of fillers resulted in a decrease in the stress at break, which was limited in the case of grafted cellulose. As an example, the stress at break of PHBV-10GC was 23% higher than PHBV-10VC. The better filler/matrix interfacial adhesion and thinner interphase observed in SEM could explain it. A better filler dispersion state could also be in favor of a limited decrease in strength at break, even if it was not clearly evidenced by SEM observations of film cross-sections.

Similarly, the strain at break dramatically decreased with filler content, especially with virgin cellulose. The decrease in elongation at break for composite with rigid fillers is explained by the fact that the proportion of stretchability is lower in composite [21]. Interestingly, this decrease was lower in the case of grafted cellulose. The grafting improved the elongation by around 30% for all the studied filler contents.

**Figure 9:**
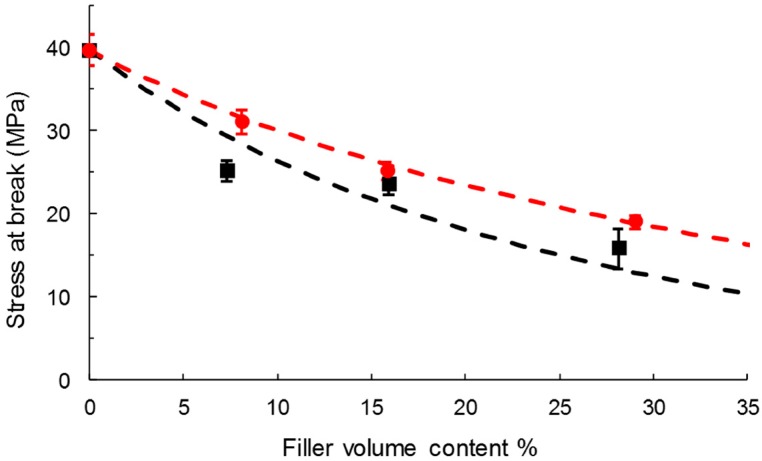
Pukánszky’s model applied for virgin cellulose (**- -,** ■), B = −0.8 and grafted cellulose (**- -, **●), B = 0.5 based composites.

As described by Pukánszky [60], an interfacial adhesion model can be used to predict the tensile strength of composites as a function of the filler content:

(10) $$\sigma_{c}=\sigma_{m}\lambda^{n}\left(\frac{1-\mathrm{xf}}{1+2.5\mathrm{xf}}\right)e^{\left(\mathrm{Bxf}\right)}$$

where σ_c_ and σ_m_ are the stress at break of the composite and matrix, respectively; xf, the filler volume fraction; and B the empirical parameter describing the quality of the filler/matrix interface. λ is relative elongation and n accounts for to the strain hardening of the matrix. Because of the small elongation of the composite, λ^n^ can be neglected [61]. The model provides information about the filler/matrix interface thanks to the parameter B: a low B corresponds to a low adhesion. In the present study, experimental data were well fitted with B = −0.8 for composites with virgin cellulose (Figure 9), confirming the poor adhesion observed in SEM. A low but positive value of B (B = 0.5) was obtained for PHBV-grafted cellulose, meaning a better interfacial adhesion. Thus, this model confirmed the idea that grafting only limited the negative impact of cellulose incorporation.

The energy at break decreased with increasing filler content (Table 4). Logically, this decrease was lower in the case of grafted cellulose. The addition of cellulose made the materials more brittle (decrease of stress and stress at break) and less tough (decrease of the energy at break). Similar results with esterified fillers in LDPE-based composites were measured by Pasquini et al. [21].

**Figure 10:**
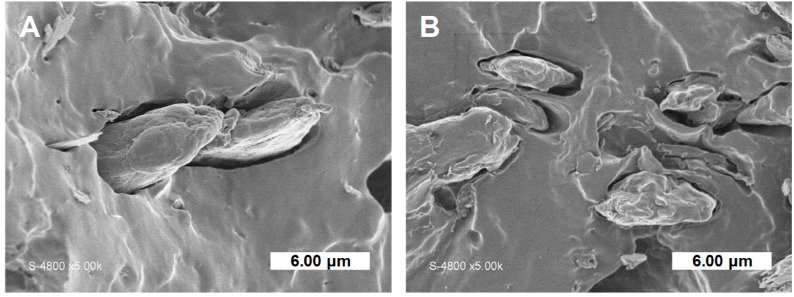
SEM pictures of fracture sections after tensile test: (**A**) PHBV-20VC and (**B**) PHBV-20CG.

SEM observations of film cross-sections after tensile tests were also carried out (Figure 10). When samples were subjected to high mechanical deformation, no difference of breaking sections between composites filled with grafted cellulose or virgin cellulose was noticed. In both cases, cellulose particles were pulled out from the matrix with the evidence of interfacial voids. Thus, when composites were mechanically solicited at high deformations, the break occurred at the filler/matrix interface even for treated cellulose. The adhesion was not sufficiently high to make the break happening in the bulk of the matrix or of the fibers.

## 4. Conclusions

In this work, we studied the structure/functional properties of biocomposites constituted of PHBV and cellulose particles either pre-treated or not. The pre-treatment consisted in gas-phase esterification using palmitoyl chloride. It was shown that gas-phase esterification of cellulosic particles allowed significant improvement to their hydrophobicity, resulting in a stronger filler/matrix interfacial adhesion and a decrease in water vapor permeability compared to virgin cellulose. The better compatibility of the esterified filler with the apolar PHBV matrix was confirmed by SEM observations of the cryo-fractured cross-sections of composite films. The gas-phase esterification of cellulose particles significantly slowed and limited the negative effects of cellulose incorporation into the composite, which offers hope for using high filler contents. However, the enhanced adhesion was not sufficient to largely improve interfacial adherence, which would be necessary to improve mechanical properties at high deformations. In conclusion, the surface grafting of cellulose particles with long aliphatic chains might be an easy and versatile tool for designing fully organic biocomposites with tailored properties.

## Figures and Tables

**Table 1:** Molecular weight (M_w_) with polydispersity index (I_p_), thermal properties (melting temperature (T_m_) and crystallization temperature (T_c_)), and degree of crystallinity (X_c_) of PHBV-based composites.

Materials	M_w_^1^ (kDa)	I_p_	T_m_^2^ (°C)	T_m_^3^ (°C)	T_c_ (°C)	X_c_^2^ (%)	X_c_^3^ (%)
PHBV	241	3.2	177 ± 1	172 ± 1	124 ± 1	63 ± 1	73 ± 1
PHBV-20VC	245	2.7	173 ± 1	171 ± 1	124 ± 1	64 ± 1	74 ± 1
PHBV-20GC	231	3.2	171 ± 3	169 ± 1	111 ± 1	59 ± 1	66 ± 1
PHBV-33VC	230	3.6	170 ± 1	169 ± 1	120 ± 1	62 ± 9	67 ± 1
PHBV-33GC	230	3.2	172 ± 4	171 ± 1	108 ± 1	69 ± 2	66 ± 1

^1^ The uncertainty was estimated at 5 kDa. ^2^ Measured at the first heating scan. ^3^ Measured at the second heating scan.

**Table 2:** Thermal degradation temperature of PHBV-based composites.

Materials	T_deg(1)_ (°C)	T_deg(2)_ (°C)	T_onset_ (°C)	T_offset_ (°C)
PHBV	297 ± 1	-	267 ± 1	312 ± 1
C-virgin	-	343 ± 1	259 ± 1	375 ± 0
C-grafted	-	336 ± 1	247 ± 1	398 ± 0
PHBV-10CV	295 ± 5	335 ± 1	248 ± 4	380 ± 4
PHBV-10CG	298 ± 1	339 ± 1	237 ± 1	413 ± 2
PHBV-20CV	292 ± 1	336 ± 4	264 ± 2	357 ± 4
PHBV-20CG	294 ± 1	340 ± 1	254 ± 1	377 ± 0
PHBV-33CV	287 ± 1	339 ± 1	256 ± 1	365 ± 1
PHBV-33CG	294 ± 1	339 ± 2	248 ± 1	389 ± 0

T_deg(1)_: Temperature of maximal degradation of the PHBV (matrix). T_deg(2)_: Temperature of maximal degradation of the cellulose (filler).

**Table 3:** Water vapor permeability (WVP), water vapor and liquid water diffusivity, and equilibrium uptake in PHBV-based biocomposites.

	Water Vapor					Liquid Water	
Materials	WVP (×10^13^ mol·m/(m^2^·s·Pa)	Apparent Diffusivity at 50% of RH (×10^−13^·m^2^·s^−1^)	Equilibrium Uptake at 50% RH (%)	Apparent Diffusivity at 95% of RH (×10^−13^·m^2^·s^−1^)	Equilibrium Uptake at 95% RH (%)	Apparent Diffusivity (×10^−13^·m^2^·s^−1^)	Equilibrium Uptake (%)
C-virgin	-	42 ± 16	8.1 ± 0.0	70.2 ± 10.1	22.7 ± 0.0	-	-
C-grafted	-	63 ± 36	6.2 ± 0.0	115 ± 62	19.0 ± 0.2	-	-
PHBV	3.7 ± 1.0	25.9 ± 6.2	0.2 ± 0.0	23.3 ± 8.7	0.5 ± 0.0	4.1 ± 0.2	0.6 ± 0.0
PHBV/20VC	5.6 ± 1.0	6.1 ± 0.6	1.0 ± 0.1	6.9 ± 2.2	3.2 ± 0.1	1.7 ± 0.1	3.7 ± 0.1
PHBV/20GC	5.0 ± 0.8	7.8 ± 0.0	0.9 ± 0.0	8.0 ± 2.7	3.3 ± 0.0	1.2 ± 0.0	3.8 ± 0.1
PHBV/33VC	36 ± 3.2	4.9 ± 0.1	2.1 ± 0.1	5.3 ± 1.2	6.6 ± 0.0	8.7 ± 0.6	6.3 ± 0.1
PHBV/33GC	15 ± 2.0	3.0 ± 0.0	1.4 ± 0.1	4.5 ± 1.2	6.1 ± 0.0	1.9 ± 0.1	5.5 ± 0.1

**Table 4:** Tensile properties (Young’s modulus, nominal stress at break, nominal strain at break, and energy at break) of PHBV-based biocomposites.

Materials	Young’s Modulus (GPa)	Stress at Break (MPa)	Strain at Break (%)	Energy at Break (mJ·cm^−3^)
PHBV	2.9 ± 0.2	39.7 ± 0.1	2.40 ± 0.15	626 ± 68
PHBV-10CV	3.2 ± 0.2	25.1 ± 1.3	0.90 ± 0.07	124 ± 15
PHBV-10CG	3.3 ± 0.1	31.0 ± 1.5	1.23 ± 0.21	229 ± 56
PHBV-20CV	3.1 ± 0.1	23.6 ± 1.3	0.91 ± 0.05	122 ± 12
PHBV-20CG	2.6 ± 0.1	25.1 ± 1.1	1.15 ± 0.05	164 ± 13
PHBV-33CV	2.6 ± 0.2	15.8 ± 2.4	0.70 ± 0.10	62 ± 17
PHBV-33CG	2.5 ± 0.2	19.0 ± 0.8	0.93 ± 0.06	101 ± 8

## References

[1] Hon D.N.S. (1994). Cellulose: A random walk along its historical path. Cellulose.

[2] Bledzki A.K., Gassan J. (1999). Composites reinforced with cellulose based fibers. Prog. Polym. Sci..

[3] Mohanty A.K., Misra M., Hinrichsen G. (2000). Biofibres, biodegradable polymers and biocomposites: An overview. Macromol. Mater. Eng..

[4] Eichhorn S.J., Baillie C.A., Zafeiropoulos N., Mwaikambo L.Y., Ansell M.P., Dufresne A., Entwistle K.M., Herrera-Franco P.J., Escamilla G.C., Groom L. (2001). Current international research into cellulosic fibres and composites. J. Mater. Sci..

[5] John M.J., Thomas S. (2008). Biofibres and biocomposites. Carbohydr. Polym..

[6] La Mantia F.P., Morreale M. (2011). Green composites: A brief review. Compos. Part A Appl. Sci. Manuf..

[7] Faruk O., Bledzki A.K., Fink H.P., Sain M. (2012). Biocomposites reinforced with natural fibers: 2000–2010. Prog. Polym. Sci..

[8] Gurunathan T., Mohanty S., Nayak S.K. (2015). A review of the recent developments in biocomposites based on natural fibres and their application perspectives. Compos. Part A Appl. Sci. Manuf..

[9] Henrique P., Pereira F., Rosa M.D.F., Odila M., Cioffi H., Cristina K., De Carvalho C., Milanese A.C., Jacobus H., Voorwald C. (2015). Vegetal fibers in polymeric composites: A review. Polimeros.

[10] Pickering K.L., Efendy M.G.A., Le T.M. (2016). A review of recent developments in natural fibre composites and their mechanical performance. Compos. Part A Appl. Sci. Manuf..

[11] Pöllänen M., Suvanto M., Pakkanen T.T. (2013). Cellulose reinforced high density polyethylene composites—Morphology, Mechanical and thermal expansion properties. Compos. Sci. Technol..

[12] Le Moigne N., Otazaghine B., Corn S., Angellier-Coussy H., Bergeret A., Navard P. (2018). Surfaces and Interfaces in Natural Fibre Reinforced Composites—Fundamentals, Modifications and Characterization.

[13] Belgacem M.N., Gandini A. (2005). The surface modification of cellulose fibres for use as reinforcing elements in composite materials. Compos. Interfaces.

[14] Trejo-O’Reilly J.-A., Cavaille J.-Y., Gandini A. (1997). The surface chemical modification of cellulosic fibres in view of their use in composite materials. Cellulose.

[15] Gauthier R., Joly C., Coupas A.C., Gauthier H., Escoubes M. (1998). Interfaces in polyolefin/cellulosic fiber composites: Chemical coupling, morphology, correlation with adhesion and aging in moisture. Polym. Compos..

[16] George J., Sreekala M.S., Thomas S. (2001). A review on interface modification and characterization of natural fiber reinforced plastic composites. Polym. Eng. Sci..

[17] Li X., Tabil L.G., Panigrahi S. (2007). Chemical treatments of natural fiber for use in natural fiber-reinforced composites: A review. J. Polym. Environ..

[18] Hubbe M.A., Rojas O.J., Lucia L.A. (2015). Green modification of surface characteristics of cellulosic materials at the molecular or nano scale: A review. BioResources.

[19] Wei L., McDonald A.G. (2016). A review on grafting of biofibers for biocomposites. Materials.

[20] Jandura P., Riedl B., Kokta B.V. (2002). Inverse gas chromatography study on partially esterified paper fiber. J. Chromatogr. A.

[21] Pasquini D., de Morais Teixeira E., da Silva Curvelo A.A., Belgacem M.N., Dufresne A. (2008). Surface esterification of cellulose fibres: Processing and characterisation of low-density polyethylene/cellulose fibres composites. Compos. Sci. Technol..

[22] Freire C.S.R., Silvestre A.J.D., Neto C.P., Gandini A., Martin L., Mondragon I. (2008). Composites based on acylated cellulose fibers and low-density polyethylene: Effect of the fiber content, degree of substitution and fatty acid chain length on final properties. Compos. Sci. Technol..

[23] Junior De Menezes A., Siqueira G., Curvelo A.A.S., Dufresne A. (2009). Extrusion and characterization of functionalized cellulose whiskers reinforced polyethylene nanocomposites. Polymer.

[24] Reulier M., Perrin R., Avérous L. (2016). Biocomposites based on chemically modified cellulose fibers with renewable fatty-acid-based thermoplastic systems: Effect of different fiber treatments. J. Appl. Polym. Sci..

[25] Yano H., Omura H., Honma Y., Okumura H., Sano H., Nakatsubo F. (2018). Designing cellulose nanofiber surface for high density polyethylene reinforcement. Cellulose.

[26] David G., Gontard N., Guerin D., Heux L., Lecomte J., Angellier-Coussy H. Gas-phase esterification of cellulose particles for the production of PHBV based biocomposites. Proceedings of the 3rd International EPNOE Junior Scientists Meeting.

[27] Berlioz S., Molina-Boisseau S., Nishiyama Y., Heux L. (2009). Gas-phase surface esterification of cellulose microfibrils and whiskers. Biomacromolecules.

[28] Fumagalli M., Ouhab D., Boisseau S.M., Heux L. (2013). Versatile gas-phase reactions for surface to bulk esterification of cellulose microfibrils aerogels. Biomacromolecules.

[29] Ku H., Wang H., Pattarachaiyakoop N., Trada M. (2011). A review on the tensile properties of natural fiber reinforced polymer composites. Compos. Part B Eng..

[30] Espert A., Vilaplana F., Karlsson S. (2004). Comparison of water absorption in natural cellulosic fibres from wood and one-year crops in polypropylene composites and its influence on their mechanical properties. Compos. Part A Appl. Sci. Manuf..

[31] Tănase E.E., Popa M.E., Râpă M., Popa O. (2015). PHB/Cellulose Fibers Based Materials: Physical, Mechanical and Barrier Properties. Agric. Agric. Sci. Procedia.

[32] Martinez-Sanz M., Vicente A., Gontard N., Lopez-Rubio A., Lagaron J.M. (2015). On the extraction of cellulose nanowhiskers from food by-products and their comparative reinforcing effect on a polyhydroxybutyrate-*co*-valerate polymer. Cellulose.

[33] Ambrosio-Martin J., Fabra M.J., Lopez-Rubio A., Gorrasi G., Sorrentino A., Lagaron J.M. (2016). Assessment of Ball Milling as a Compounding Technique to Develop Nanocomposites of Poly(3-Hydroxybutyrate-*co*-3-Hydroxyvalerate) and Bacterial Cellulose Nanowhiskers. J. Polym. Environ..

[34] Wolf C., Guillard V., Angellier-Coussy H., Silva G.G.D., Gontard N. (2016). Water vapor sorption and diffusion in wheat straw particles and their impact on the mass transfer properties of biocomposites. J. Appl. Polym. Sci..

[35] Bhardwaj R., Mohanty A.K., Drzal L.T., Pourboghrat F., Misra M. (2006). Renewable Resource-Based Green Composites from Recycled Cellulose Fiber and Poly (3-hydroxybutyrate-*co*-3-hydroxyvalerate) Bioplastic. Biomacromolecules.

[36] Jiang L., Huang J., Qian J., Chen F., Zhang J., Wolcott M.P., Zhu Y. (2008). Study of poly(3-hydroxybutyrate-*co*-3-hydroxyvalerate) (PHBV)/bamboo pulp fiber composites: Effects of nucleation agent and compatibilizer. J. Polym. Environ..

[37] Yu H., Qin Z. (2012). Effect of Cellulose nanocrystal on Crystallization Behavior of Poly (3-hydroxybutyrate-*co*-3-hydroxyvalerate). Adv. Mater. Res..

[38] Ten E., Jiang L., Wolcott M.P. (2012). Crystallization kinetics of poly (3-hydroxybutyrate-*co*-3-hydroxyvalerate)/cellulose nanowhiskers composites. Carbohydr. Polym..

[39] Srithep Y., Ellingham T., Peng J., Sabo R., Clemons C., Turng L., Pilla S. (2013). Melt compounding of poly (3-hydroxybutyrate-*co*-3-hydroxyvalerate)/nano fibrillated cellulose nanocomposites. Polym. Degrad. Stab..

[40] Yu H., Yan C., Yao J. (2014). Fully biodegradable food packaging materials based on functionalized cellulose nanocrystals/poly(3-hydroxybutyrate-*co*-3-hydroxyvalerate) nanocomposites. RSC Adv..

[41] Sánchez-Safont E.L., González-Ausejo J., Gámez-Pérez J., Lagarón J.M., Cabedo L. (2016). Poly(3-Hydroxybutyrate-*co*-3-Hydroxyvalerate)/Purified Cellulose Fiber Composites by Melt Blending: Characterization and Degradation in Composting Conditions. J. Renew. Mater..

[42] Malmir S., Montero B., Rico M., Barral L., Bouza R. (2017). Morphology, thermal and barrier properties of biodegradable films of poly (3-hydroxybutyrate-*co*-3-hydroxyvalerate) containing cellulose nanocrystals. Compos. Part A.

[43] Thoury-Monbrun V., Angellier-Coussy H., Guillard V., Legland D., Gaucel S. (2018). Impact of two-dimensional particle size distribution on estimation of water vapor diffusivity in micrometric size cellulose particles. Materials.

[44] Barham P.J., Keller A., Otun E.L., Holmes P.A. (1984). Crystallization and morphology of a bacterial thermoplastic: Poly-3-hydroxybutyrate. J. Mater. Sci..

[45] Thoury-Monbrun V. (2018). Formalisation des relations structure/propriétés de transfert de matière dans un biocomposite modèle. Ph.D. Thesis.

[46] Crank J. (1975). The Mathematics of Diffusion.

[47] Spitalsky Z., Lacik I., Lathova E., Janigova I., Chodak I. (2006). Controlled degradation of polyhydroxybutyrate via alcoholysis with ethylene glycol or glycerol. Polym. Degrad. Stab..

[48] Berthet M.A., Angellier-Coussy H., Machado D., Hilliou L., Staebler A., Vicente A., Gontard N. (2015). Exploring the potentialities of using lignocellulosic fibres derived from three food by-products as constituents of biocomposites for food packaging. Ind. Crops Prod..

[49] Berthet M.A., Mayer-Laigle C., Rouau X., Gontard N., Angellier-Coussy H. (2017). Sorting natural fibres: A way to better understand the role of fibre size polydispersity on the mechanical properties of biocomposites. Compos. Part A Appl. Sci. Manuf..

[50] Lammi S., Le Moigne N., Djenane D., Gontard N., Angellier-Coussy H. (2018). Dry fractionation of olive pomace for the development of food packaging biocomposites. Ind. Crops Prod..

[51] Banik G., Brückle I. (2010). Principles of Water Absorption and Desorption in Cellulosic Materials. Restaur. Int. J. Preserv. Libr. Arch. Mater..

[52] Bugnicourt E., Cinelli P., Lazzeri A., Alvarez V. (2014). Polyhydroxyalkanoate (PHA): Review of synthesis, characteristics, processing and potential applications in packaging. Express Polym. Lett..

[53] Marais S., Nguyen Q.T., Devallencourt C., Metayer M., Nguyen T.U., Schaetzel P. (2000). Permeation of water through polar and nonpolar polymers and copolymers: Determination of the concentration-dependent diffusion coefficient. J. Polym. Sci. Part B Polym. Phys..

[54] Shogren R. (1997). Water vapor permeability of biodegradable polymers. J. Environ. Polym. Degrad..

[55] Tomé L.C., Pinto R.J.B., Trovatti E., Freire C.S.R., Silvestre A.J.D., Neto C.P., Gandini A. (2011). Transparent bionanocomposites with improved properties prepared from acetylated bacterial cellulose and poly(lactic acid) through a simple approach. Green Chem..

[56] Avella M., La Rota G., Martuscelli E., Raimo M., Sadocco P., Elegir G., Riva R. (2000). Poly(3-hydroxybutyrate-*co*-3-hydroxyvalerate) and wheat straw fibre composites: Thermal, mechanical properties and biodegradation behaviour. J. Mater. Sci..

[57] Dufresne A., Dupeyre D., Paillet M. (2003). Lignocellulosic Flour-Reinforced Poly(hydroxybutyrate-*co*-valerate) Composites. J. Appl. Polym. Sci..

[58] Singh S., Mohanty A.K. (2007). Wood fiber reinforced bacterial bioplastic composites: Fabrication and performance evaluation. Compos. Sci. Technol..

[59] Ludueña L., Vázquez A., Alvarez V. (2012). Effect of lignocellulosic filler type and content on the behavior of polycaprolactone based eco-composites for packaging applications. Carbohydr. Polym..

[60] Pukánszky B. (1990). Influence of interface interaction on the ultimate tensile properties of polymer composites. Composites.

[61] Dányádi L., Janecska T., Szabó Z., Nagy G., Móczó J., Pukánszky B. (2007). Wood flour filled PP composites: Compatibilization and adhesion. Compos. Sci. Technol..

